# Positive impact of retrograde autologous priming in adult patients undergoing cardiac surgery: a randomized clinical trial

**DOI:** 10.1186/s13019-018-0739-0

**Published:** 2018-05-21

**Authors:** Britt Hofmann, Claudia Kaufmann, Markus Stiller, Thomas Neitzel, Andreas Wienke, Rolf-Edgar Silber, Hendrik Treede

**Affiliations:** 1 Department of Cardiac Surgery, University Hospital Halle, Ernst-Grube-Strasse 40, 06120 Halle, Germany; 2 Institute of Medical Epidemiology, Biostatistics and Informatics, Martin-Luther-University Halle-Wittenberg, 06097 Halle, Germany

**Keywords:** Retrograde autologous priming (RAP), Cardiac surgery, Perioperative management, Blood transfusion

## Abstract

**Background:**

Adult cardiac surgery with extracorporeal circulation is known to be associated with increased risk of blood transfusion leading to adverse outcomes. Procedures like retrograde autologous priming (RAP) may reduce these negative side effects. This randomized prospective study was initiated to assess whether RAP using specifically designed RAP bag (Terumo) has immediate effects on patient outcome.

**Methods:**

One hundred eighteen adults undergoing elective CABG or elective aortic valve replacement were randomly assigned by a computer program into two groups: the RAP group (*n* = 54) in which the retrograde autologous priming was applied and the non-RAP (*n* = 64) group in which the same setting was used without the possibility to save priming volume. Patient demographics, preoperative characteristics and postoperative outcomes were analyzed for both groups.

**Results:**

The primary endpoint defined as rate of intraoperative blood transfusion was significantly reduced in the RAP-group (*p* = 0.04). The absolute risk reduction for RAP managed patients was 13.5 percent points. There were no significant differences in operation time and blood loss. No deaths and no myocardial infarctions were observed. The number of patients needed to treat to prevent at least one red blood cell transfusion was around 8 (NNT = 7.42).

**Conclusions:**

Retrograde autologous priming is a safe and less invasive procedure which achieves clear benefits for adult cardiac surgery patients. In the light of increasing red blood cell transfusion risks and costs and the wish of patients to avoid a transfusion implementation of retrograde autologous priming is an interesting option.

**Trial registration:**

German Clinical Trials Register ID: DRKS00013512, registered 04 December 2017.

## Background

Despite notable advances in extracorporeal circulation during the last decade, cardiac surgery with cardiopulmonary bypass (CPB) is still associated with an increased risk of blood transfusions [1]. Today, using the gold-standard technique of CPB, elective coronary artery revascularization and aortic valve replacement can be performed with a mortality rate of less than 3% (https://iqtig.org/berichte/strukturierter-qualitaetsbericht/2015/). The primary setup of the CPB circuit demands a priming volume of approximately 1500 mL of crystalloid solution [2], which leads to a relevant hemodilution. Hemodilution resulting in low hematocrit levels during CPB is known to be responsible for impaired hemostasis, detrimental effects on end-organ function and on cognitive outcome [3–5]. In consequence, nearly 50% of all cardiac surgery patients [6] receive a transfusion of red blood cells and cardiac surgery accounts for a significant amount of blood product consumption worldwide [7]. Blood transfusions have been associated with several serious complications, like transfusion related acute lung injury, modulation of the immune system and increased post-operative infection risk [6]. Furthermore, blood transfusions are an independent risk factor for morbidity and mortality after cardiac surgery [8] and responsible for considerable healthcare costs [9–11]. Therefore, the Society of Thoracic Surgeons and the Society of Cardiovascular Anesthesiologists guidelines recommend efforts to reduce blood transfusion in cardiac surgery [12]. The measures employed in our institution to reduce hemodilution intraoperatively include cell salvage procedures, reduction of CPB priming volume and retrograde autologous priming (RAP). RAP was first introduced in 1959 by Panico and Neptune [13], in 1998 Rosengart et al. [14] refined and reintroduced the technique in clinical practice. The modern RAP procedure minimizes hemodilution by displacing the crystalloid priming volume of arterial and venous lines via passive exsanguination of native blood prior to CPB initiation. Procedures like retrograde autologous priming may further reduce the negative side effects of ECC and transfusion related healthcare costs. Several studies focusing on RAP have documented varying effectiveness in reducing transfusion rates and equivalent or improved outcomes associated with this technique [7, 14–19]. This randomized prospective study was initiated to assess whether RAP using specifically designed RAP bag (TERUMO, Europe NV, Leuven, Belgium) has immediate effects on hemodilution, blood transfusions and patient outcome. Our study is the first randomized trial were the effect measure number needed to treat for clinical decision making was calculated. Furthermore, we focused on cost-effectiveness of the RAP procedure, as well as on other factors predicting postoperative blood transfusions.

## Methods

### Study patients

**Fig. 1:**
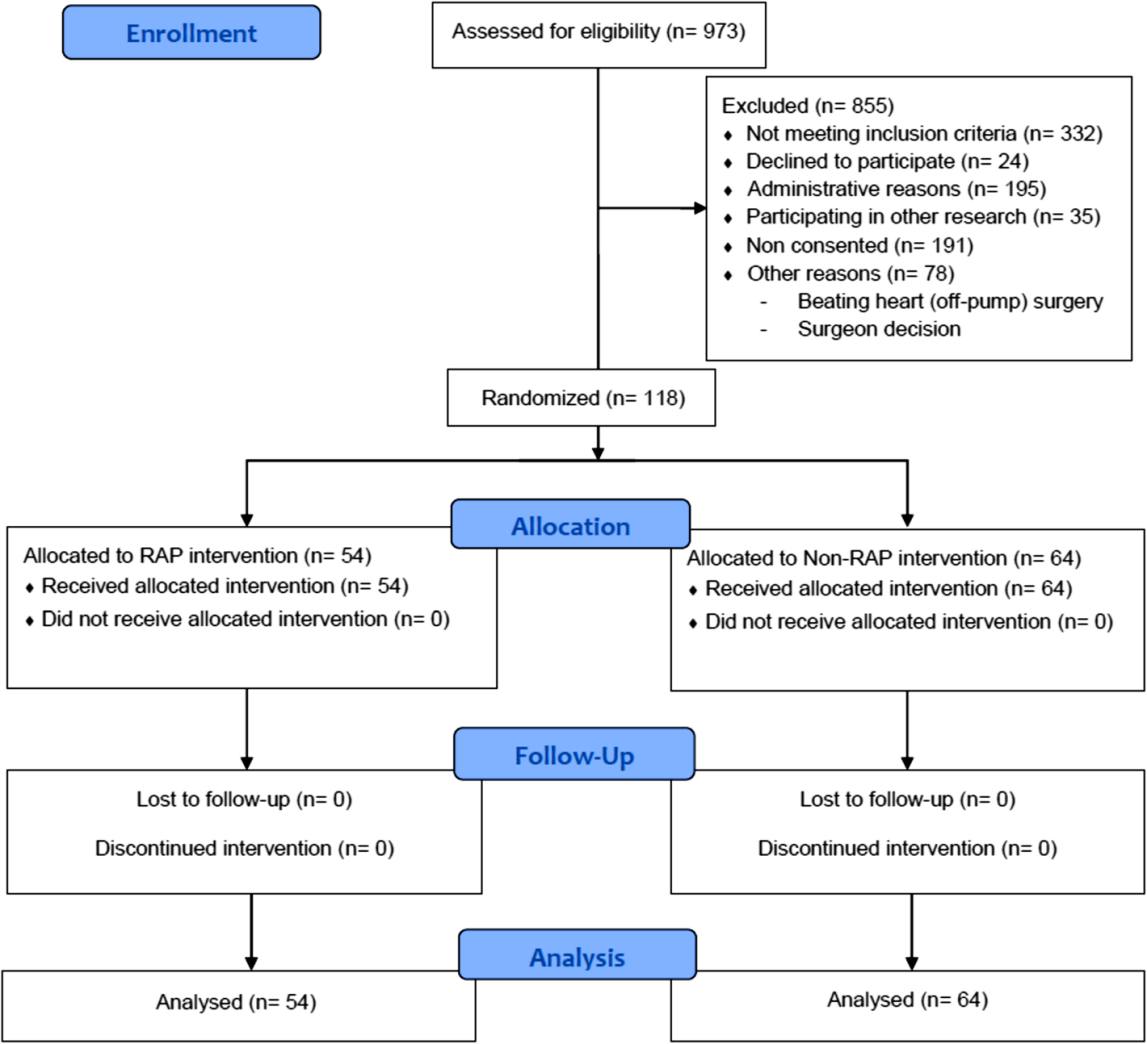
CONSORT flow diagram (RAP = retrograde autologous priming technique)

In the present study, 118 adults undergoing first-time elective CABG or elective aortic valve replacement between August 2012 and July 2015 were randomly assigned by a computer program into two groups: the RAP group (*n* = 54) in which the retrograde autologous priming was applied and the non-RAP (*n* = 64) group in which the same setting was used without the possibility to save priming volume (Fig. 1). All patients were admitted to our unit the day before the planned operation. All patients received aspirin 100 mg/d until the day before the operation. Exclusion criteria were age < 18 years, LVEF ≤ 20%, emergency operations, reoperations, combined procedures, myocardial infarction 24 h before surgery, preoperative cortisone, coumarin, dual platelet inhibitor, or IV heparin therapy, thrombocytopenia (< 100 Gpt/l), liver disease, preoperative dialysis, hematological or oncological systemic disease or systemic infection. As an investigator initiated project, the trial was registered as RAP 18080 at the local research portal of the federal state of Saxony-Anhalt (https://forschung-sachsen-anhalt.de/) on August 18th, 2012.

### Randomization

Consenting patients meeting inclusion criteria were randomly assigned at the day of surgery. The randomization was computerized by software. This software generated a binary result by chance. Each was strictly associated with one study group and was linked to the subject’s ID. The ID, the time of randomization, and the assigned study group were stored in a MySQL database.

### Anticoagulation management

The Heparin concentration management system has been used in our institution since the year 2010 and we developed a protocol for the rational use of the device. Both study groups were managed according to this protocol [20]. In all patients, tranexamic acid (Cyklokapron®) was used as a standard perioperative bleeding prophylaxis to prevent hyperfibrinolysis. According to our institutional standards all patients received a cumulative dosage of 4 g tranexamic acid.

### Anesthesia and cardiopulmonary bypass circuit

Anesthesia was initiated using an intravenous technique with propofol, fentanyl and pancuronium bromide on demand. After initiation anaesthesia was performed as balanced anesthesia, administered as low fresh gas flow with inhalational sevoflurane over the ventilator or the oxygenator of the CPB system; additional fentanyl was administered intravenously. Normothermic extracorporeal circulation was accomplished in all patients with an open non-heparin-coated system including a poly-2-methoxyethyl-acrylate (PMEA) coated hollow-fiber membrane oxygenator (CAPIOX® FX15) with integrated arterial filter, and reservoir (Terumo). The CPB system was primed with 850 ml Jonosteril® and 10.000 IU of porcine heparin. Management of CPB included systemic temperature of 36 °C, α-stat pH management, target mean perfusion pressure between 50 and 70 mmHg, and pump flow rates of ≥ 2.4 l/min/m^2^. Myocardial protection was achieved with intermittent antegrade warm cardioplegia according to Calafiore’s protocol [21]. All patients received an intraoperative infusion of tranexamic acid (bolus of 2 g after the administration of the initial heparin bolus and another 2 g were given into the CPB circuit during reperfusion). In all patients, a cell saver (Medtronic autoLog™ or Sorin Xtra®) was used to collect wound blood prior to CPB, blood from the pleural space and CPB volume, which could not be reinfused before protamine administration. This blood was processed if the collected volume exceeded 600 ml.

### Retrograde autologous priming

The only structural difference to the standard CPB circuit is a RAP bag (CE, Terumo, Belgium). Before RAP started, mean arterial pressure was elevated to approximately 80 mmHg using small doses of i.v. noradrenaline. Then priming fluid in the arterial line of the heart-lung-machine was displaced with patient’s blood using arterial pressure, the venous line was drained by slowly rotating the arterial pump. The priming fluid of the CPB circuit was slowly drained into the RAP bag. The RAP bag stayed connected with the venous reservoir for crystalloid fluid replacement during CPB. The retrograde priming procedure took approximately 3 to 5 min.

### Transfusion guidelines

Packed red blood cells (PRB) were transfused when hemoglobin concentration (Hb) was < 7.0 g/dl or hematocrit (Hk) < 21% during CPB, and after cardiac surgery if Hb < 8.0 g/dl or Hk < 24% [22–24]. The transfusion of fresh frozen plasma (FFP) and platelet concentrates was based on the clinical decision of the anesthetist. According to the departmental protocol a hemorrhage of over 1000 ml within the first 6 postoperative hours was an indication for surgical re-exploration.

### Data collection

Patient demographics, preoperative characteristics and postoperative outcomes were analyzed for both groups. For all patients, more than 200 variables were entered in a database. All clinical laboratory parameters were assessed by the central department of Laboratory Medicine of the University Hospital.

### Study endpoints

The primary endpoint for this study was determined as intraoperative blood transfusion. Other endpoints were in-hospital blood transfusion (intraoperative or postoperative), defined safety criteria like intra- and postoperative complications (renal failure, stroke, prolonged ventilation, reintubation, ICU stay, in-hospital stay, death).

### Statistical analyses

Categorical variables were expressed as frequencies and percentages. Metric variables were expressed as mean ± standard deviation (SD). The benefit of the RAP use over the control was tested with the chi-square test and was further expressed as the absolute risk reduction (ARR). For clinical decision making the effect measure number needed to treat (NNT) was calculated [25]. To predict risk factors for intraoperative and in-hospital blood transfusion, simple and multivariable logistic regression analyses were done to estimate the odds ratios (OR) with their 95% confidence intervals (CI). Discrimination was assessed using the area under the receiver operating characteristic curve (AUROC). AUROC analysis was also utilized to calculate cut off values. Sample size calculation was based on a two-sided chi-square-test with significance level alpha = 5%, a power of 90% and expected intraoperative transfusion rates of 5% in the RAP group and 25% in the non-RAP group, respectively, and resulted in *n* = 65 patients per group. However, the study had to be terminated due to relevant changes in value measurement procedures of the central department of Laboratory Medicine. Especially, the change from troponin I to troponin T measurement as well as the change of the measurement methods for kidney, liver, hematological and hemolysis parameters was critical. Because it was not possible to establish a trouble-free conversion, we felt that the clear analysis of the study was too heavily biased, so we decided to terminate the enrolment. The authors did not look into the data before terminating the enrolment.

## Results

### Patients

In the present prospective randomized study, we assessed 118 patients (mean age: 69.74 ± 8.74 years, range 42–87 years). Eighteen (15.25%) of the 118 patients were females and they were slightly older (70.3 ± 7.1 years) than males (69.6 ± 9 years). The pre-operative left ventricular ejection fraction was normal with 58.9 ± 10.3% in the overall patient population. Further demographic and clinical data of the study individuals are shown in Table 1. There was no clinically relevant difference between the two groups with exception for BMI and hematocrit. Most patients had comorbidities and risk factors for coronary artery disease. All patients had first time cardiac surgery. Surgical details are shown in Table 2. Ninety-five patients underwent isolated coronary artery bypass grafting (80%), 21 patients underwent isolated aortic valve surgery (18%) and 2 patients underwent combined single CABG and aortic valve surgery (2%). The two patients with combined procedures were initially planned for aortic valve replacement. However, in both cases the surgeon decided intraoperatively, that the patients would profit from a venous bypass graft (one case CABG-D1, other case CABG-RCA). Both patients were in the RAP group. There were no other differences between the groups regarding the type of operation or other surgical details, including the number of bypass grafts performed and the cardiopulmonary bypass, aortic cross-clamp and total surgical times (Table 2). RAP could reduce the priming volume of the ECC by 357 ± 57 mL.

**Table 1:** Baseline characteristics of study patients

Variables	RAP patients (*n* = 54)	Non-RAP (*n* = 64)
Age (years)	69.94 ± 8.65	69.56 ± 8.90
Male gender	81.48 (44/54)	87.50 (56/64)
Height (cm)	172.04 ± 8.12	172.44 ± 7.32
Weight (kg)	87.52 ± 16.07	83.16 ± 12.42
BMI (kg/m^2^)	29.57 ± 5.36	27.88 ± 3.52
Hypertension	92.59 (50/54)	95.31 (61/64)
Diabetes mellitus	38.89 (21/54)	40.63 (26/64)
Diabetes Type		
IDDM	33.33 (7/21)	23.08 (6/26)
NIDDM	66.67 (14/21)	76.92 (20/26)
Hypercholesterolemia	79.63 (43/54)	79.69 (51/64)
Renal disease	16.67 (9/54)	15.63 (10/64)
Smoking Status		
Currently	12.96 (7/54)	20.31 (13/64)
Never	64.81 (35/54)	65.63 (42/64)
Previously	22.22 (12/54)	14.06 (9/64)
Chronic Lung Disease	7.41 (4/54)	17.19 (11/64)
PAD	16.67 (9/54)	4.69 (3/64)
Atrial Fibrillation	11.11 (6/54)	7.81 (5/64)
NYHA class		
I	11.32 (6/53)	11.48 (7/61)
II	43.40 (23/53)	52.46 (32/61)
III	43.40 (23/53)	31.15 (19/61)
IV	1.89 (1/53)	4.92 (3/61)
CCS class		
I	16.67 (6/36)	14.89 (7/47)
II	44.44 (16/36)	46.81 (22/47)
III	38.89 (14/36)	38.30 (18/47)
LVEF (%)	59.93 ± 10.37	57.95 ± 10.16
CAD		
1 vessel CAD	20.37 (11/54)	17.19 (11/64)
2 vessel CAD	22.22 (12/54)	14.06 (9/64)
3 vessel CAD	57.41 (31/54)	68.75 (44/64)
Aortic valve stenosis	24.07 (13/54)	15.63 (10/64)
Baseline Hct (%)	37.7 ± 3.9	38.2 ± 4.1
Baseline creatinine (μmol/L)	79.00 ± 19.07	76.84 ± 19.10
Logistic EuroSCORE	3.81 ± 2.21	3.41 ± 2.51
EuroSCORE II	1.44 ± 0.70	1.27 ± 0.88

Values are mean ± SD or n (%). *Baseline Hct* preoperative hematocrit, *BMI* body mass index, *CAD* Coronary artery disease, *CABG* coronary artery bypass grafting, *CCS class* Canadian Cardiovascular Society classification, *IDDM* insulin dependent diabetes mellitus, *NIDDM* non-insulin dependent diabetes mellitus, *NYHA class* New York Heart Association classification, *LVEF* left ventricular ejection fraction, *PAD* peripheral artery disease

**Table 2:** Relevant intraoperative details of study patients

Variables	RAP patients (*n* = 54)	Non-RAP (*n* = 64)
CABG distal Anastomoses	2.41 ± 1.38	2.63 ± 1.33
AVR	24.07 (13/54)	15.63 (10/64)
Total surgical procedure (min)	172.41 ± 27.48	175.09 ± 35.00
Cardiopulmonary bypass (min)	84.98 ± 14.08	87.13 ± 22.60
Aortic cross-clamp time (min)	52.67 ± 12.06	52.16 ± 14.78
Reperfusion (min)	25.15 ± 7.76	26.19 ± 9.81
Total crystalloid volume (mL)	2065.74 ± 978.97	1942.19 ± 1142.27
Plasma expanders administered	61.11 (33/54)	53.13 (34/64)
Cellsaver volume harvest (mL)	710.22 ± 538.21	659.77 ± 541.88
Cellsaver volume wash (mL)	163.87 ± 245.51	146.97 ± 235.04
MAP (mmHg)	75.8 ± 5.1	77.6 ± 4.7
Adrenalin dose (μg/kg/min)	0.005 ± 0.02	0.009 ± 0.03
Noradrenalin dose (μg/kg/min)	0.06 ± 0.06	0.06 ± 0.05

Values are mean ± SD or n (%). *AVR* aortic valve replacement, *MAP* mean arterial pressure

### Primary endpoint

The intraoperative transfusion rate was 3.7% (2 of 54 patients) in the RAP group and 17.2% (11 of 64 patients) in the non-RAP group. The primary endpoint defined as rate of intraoperative blood transfusion was significantly reduced in the RAP group (*p* = 0.04). The absolute risk reduction for RAP managed patients was 13.5 percent points. Non-RAP managed patients had an increased relative risk for an intraoperative red blood cell transfusion of 4.6 (95% CI, 1.07 to 20.03; *p* = 0.039) compared to RAP managed patients. The number of patients needed to treat to prevent at least one red blood cell transfusion was around 8 (NNT = 7.42). With regard to the fact, that in most of the cases two red blood cell concentrates (costs per RCC: 272 € [11]) are administered, the RAP procedure (costs per RAP bag: 60 €) with 480 € costs for the treatment of eight patients versus 544 € costs for two RCC is cost-effective.

### Additional analyses

As we noticed an imbalance between the two patient groups regarding body mass index and preoperative hematocrit value, we adjusted for these potential confounders in a multivariable logistic regression analysis for the primary endpoint intraoperative blood transfusion (Table 3).

**Table 3:** Multivariable logistic regression analysis for intraoperative blood transfusion

Variables	Multivariable Logistic Regression		
	OR	95%-CI	p
BMI (kg/m^2^)	0.99	0.84–1.16	0.88
Baseline Hct (%)	0.75	0.62–0.91	0.003
Non-RAP	6.93	1.34–35.74	0.02

*OR* Odds Ratio, *95%-CI* 95%- confidence interval for OR, *BMI* body mass index, *Hct* hematocrit, *RAP* retrograde autologous priming

### Secondary endpoints and additional analyses

Also, we did a multivariable logistic regression analysis for in-hospital blood transfusion and adjusted for the potential confounders body mass index, preoperative hematocrit value and blood loss 12 h post-operative (Table 4). As independent predictors of the outcome blood transfusion throughout the hospital stay we identified a body mass index > 29 kg/m^2^ (AUROC ± SE, 0.68 ± 0.05; 95%-CI, 0.58–0.76; *p* = 0.0007), a preoperative hematocrit value ≤ 36% (AUROC ± SE, 0.8 ± 0.04; 95%-CI, 0.72–0.87; *p* <  0.0001), blood loss 12 h post-operative > 450 mL (AUROC ± SE, 0.63 ± 0.05; 95%-CI, 0.54–0.72; *p* = 0.01) and RAP ≤ 350 mL (AUROC ± SE, 0.7 ± 0.08; 95%-CI, 0.54–0.81; *p* = 0.01).

**Table 4:** Multivariable logistic regression analysis for in-hospital blood transfusion

Variables	Multivariable Logistic Regression		
	OR	95%-CI	p
BMI (kg/m^2^)	1.26	1.11–1.42	0.0004
Baseline Hct (%)	0.62	0.52–0.75	**<** 0.0001
Blood loss 12 h post-op. (mL)	1.01	1.002–1.01	0.008
Non-RAP	3.38	1.13–10.12	0.03

*OR* Odds Ratio, *95%-CI* 95%- confidence interval for OR, *BMI* body mass index, *Hct* hematocrit, *RAP* retrograde autologous priming

The postoperative MAP was 79.8 ± 9.7 mmHg in the RAP group and 81.2 ± 9.2 mmHg in the Non-RAP group. The noradrenalin dose up to 16 h postoperative was 2505 ± 3087 μg in RAP patients and 2465 ± 2929 μg in Non-RAP patients. Postoperative adrenalin was used in 7.41% (4/54; 195.3 ± 745.3 μg) of RAP and 12.5% (8/64; 604.4 ± 1989.8 μg) of Non-RAP managed patients.

Data on postoperative complications are shown in Table 5. There were no perioperative deaths, defined as a death within 30 days of surgery or prior to discharge following surgery.

**Table 5:** Postoperative complications of the study patients

Postoperative complication	RAP patients (*n* = 54)	Non-RAP (*n* = 64)	*p* value
Prolonged ventilation > 48 h	0	4.69 (3/64)	0.31
Re-intubation	1.85 (1/54)	4.69 (3/64)	0.74
Bleeding	7.41 (4/54)	4.69 (3/64)	0.81
Myocardial infarction	0	0	
Reoperation	0	0	
Renal failure	0	0	
Stroke	0	0	
Mediastinitis	0	0	
Perioperative death	0	0	
Length of stay in ICU (d)	2.02 ± 2.8	2.3 ± 2.6	0.57
Length of in-hospital stay (d)	15.4 ± 4.75	15.02 ± 6.4	0.72

Values are mean ± SD or n (%)

## Discussion

In the present study, we could show that retrograde autologous priming is a safe, simple to use and effective procedure to reduce blood transfusions in elective adult cardiac surgery. RAP managed patients had a significantly reduced rate of intraoperative red blood cell transfusions, the number of patients needed to treat with RAP to prevent one red blood cell transfusion was around 8. With regard to the fact, that in most of the cases two red blood cell concentrates are administered, the RAP procedure with a cost of 480 € for the treatment of eight patients versus 544 € costs for two red blood cell concentrates [11] is also cost-effective. A major concern with RAP is the possible need for vasopressor support during volume reduction. However, we noticed that this is only transient with no long-term impact for patients. In this randomized study, the RAP technique was performed safely in patients undergoing ECC without adding evident additional time to the procedure.

Further analyses revealed a body mass index over 29 kg/m^2^, a preoperative hematocrit value of ≤ 36% and a 12 h postoperative blood loss of over 450 mL as independent predictors for in-hospital blood transfusion after elective adult cardiac surgery. To be effective in avoiding in-hospital blood transfusion the RAP volume had to be at least 350 mL. We did not find a difference in postoperative complications or operative mortality between groups.

A low baseline hematocrit was identified as risk factor for intraoperative transfusion and was an independent predictor for in-hospital blood transfusion in general. According to the new 2017 EACTS/EACTA Guidelines on patient blood management for adult cardiac surgery [26, 27], 48% of our RAP managed patients and 45% of the control group had a mild anemia (women, Hb 100–120 g/L; men, Hb 100–130 g/L). For future optimal preoperative management of red blood cells in line with the guidelines and available data [28, 29] we need to elucidate reasons for preoperative anemia (e.g. iron deficiency, vitamin D or folate deficiency) and implement erythropoietin (EPO) treatment with or without iron supplementation (class IIa, level B recommendation) in our subsequent work.

The results of our randomized trial are in line with data reported by Rosengart et al. [14] and current data from Teman et al. [7], Trapp et al. [19] and a meta-analysis by Sun et al. [30]. This growing body of data supports the findings of our study that RAP decreases intraoperative and postoperative blood transfusion rates without increasing peri- or postoperative complications, leading to comparable or even favorable outcomes. Our study is the first randomized trial were the effect measure number needed to treat for clinical decision making was calculated, showing that eight patients need to be treated with RAP to avoid at least one intraoperative blood transfusion. We also showed for the first time that a minimum RAP volume of 350 mL is needed for the procedure to be effective. Furthermore, known factors influencing the transfusion rate, such as the preoperative hematocrit, were confirmed. Despite these results the use of RAP technique in adult cardiac surgery has only been given a level II B recommendation from the practice guidelines of the Society of Thoracic Surgeons and the Society of Cardiovascular Anesthesiologists [12]. Considering all the reported data and the findings of the current study confirming safety and efficacy of the technique, RAP should potentially be used in all patients undergoing cardiopulmonary bypass. With regard to Likosky et al. [1], we also feel that blood conservation in cardiac surgery needs more efforts and a team approach. Our goal should be to avoid considerable hemodilution due to ECC, especially in the growing body of patients with high body mass index and low preoperative hematocrit. We are convinced that particularly in these patients saving blood products by avoiding hemodilution will have huge consequences regarding postoperative infection rates and morbidity. This will be implemented within our subsequent work.

### Study limitations

Our study is subject to the limitations inherent in studies from a single center. Furthermore, the treatment group variable (non-RAP vs. RAP) is associated with large confidence intervals (Tables 3 and 4). This is mainly a consequence of the binary nature of this variable compared to the more informative continuous variables BMI, baseline hematocrit, and blood loss. Additionally, because of the small sample population and numbers of complications, the uses of body mass index, preoperative hematocrit value, blood loss 12 h post-operative and RAP as predictors for in-hospital blood transfusions in cardiac surgery require further evaluation.

## Conclusions

In conclusion, this randomized study observed in non-RAP managed patients an increased relative risk for intraoperative red blood cell transfusion. The number of patients needed to treat with RAP to prevent one red blood cell transfusion was around 8. To our knowledge, this is the first study to confirm that RAP is cost-effective relating to intraoperative blood transfusions. In addition, our data revealed RAP, BMI, preoperative hematocrit and 12 h postoperative blood loss as independent predictors for in-hospital blood transfusions after elective adult cardiac surgery. Adopting the RAP technique into the daily perfusion routine does not require sophisticated or expensive pharmacologic or technical modifications and is not related to increased peri- or postoperative patient risks. Therefore, we recommend considering this method in adult patients scheduled for elective cardiac surgery.

## Abbreviations

AUROC: Area under the receiver operating characteristic curve
BMI: Body mass index
CI: Confidence interval
CPB: Cardiopulmonary bypass
ECC: Extracorporeal circulation
NNT: Number of patients needed to treat
RAP: Retrograde autologous priming
RCC: Red blood cell concentrates
SD: Standard deviation
SE: Standard error
